# Changing dynamics of bloodstream infections due to methicillin-resistant *Staphylococcus aureus* and vancomycin-resistant *Enterococcus faecium* in Germany, 2017–2023: a continued burden of disease approach

**DOI:** 10.1186/s13756-025-01522-9

**Published:** 2025-01-30

**Authors:** Simon Brinkwirth, Marcel Feig, Ines Noll, Tim Eckmanns, Achim Dörre, Sebastian Haller, Niklas Willrich

**Affiliations:** 1 Unit 37: Healthcare-Associated Infections, Surveillance of Antibiotic Resistance and Consumption, Department of Infectious Disease Epidemiology, Robert Koch Institute, Seestraße 10, 13353 Berlin, Germany; 2 Department of Infectious Disease Epidemiology, Postgraduate Training for Applied Epidemiology (PAE), Robert Koch Institute, Seestraße 10, 13353 Berlin, Germany; 3 ECDC Fellowship Programme, Field Epidemiology Path (EPIET), European Centre for Disease Prevention and Control (ECDC), Stockholm, Sweden; 4 Unit MF2: Domain Specific Data Competence Centre, Department of Methods Development, Research Infrastructure and Information Technology, Robert Koch Institute, Seestraße 10, 13353 Berlin, Germany

## Abstract

**Background:**

Antimicrobial resistance is a global threat to public health, with methicillin-resistant *Staphylococcus aureus* (MRSA) and vancomycin-resistant *Enterococcus faecium* (VREfm) being major contributors. Despite their clinical impact, comprehensive assessments of changes of the burden of bloodstream infections in terms of Disability-Adjusted Life Years (DALYs) and attributable deaths over time are lacking, particularly in Germany.

**Methods:**

We used data from the Antimicrobial Resistance Surveillance system, which covered about 30% of German hospitals. Bloodstream infections were defined by a VREfm or MRSA-positive blood culture. We estimated incidences as a first step to further use these rates to calculate DALYs and attributable deaths using the Burden of Communicable Disease in Europe toolkit. The analysis included stratification by age, sex and region.

**Results:**

From 2017 to 2023, 6262 MRSA and 5442 VREfm blood culture-positive isolates were identified. The incidence of MRSA bloodstream infections decreased from 4.0 to 2.1 per 100,000 population, with estimated DALYs decreasing from 14.6 to 8.6 per 100,000 and attributable deaths from 591 to 316. Conversely, VREfm-BSI incidence doubled from 1.7 to a peak of 3.0 (2021) before declining back to 1.7 per 100,000 in 2023, with estimated DALYs increasing from 8.9 to 16.5 and then decreasing to 8.5 per 100,000 and attributable deaths increasing from 317 to 327. Men and people over 60 years had the highest burden, with noticeable regional differences.

**Conclusion:**

MRSA and VREfm bloodstream infections followed different trends in the past and now present a comparable burden in Germany. Both pathogens pose a significant threat, particularly to hospitalised older aged men. Our findings highlight the need for targeted prevention and continued surveillance of MRSA and VREfm to reduce infections and their impact.

**Supplementary Information:**

The online version contains supplementary material available at 10.1186/s13756-025-01522-9.

## Introduction

Antimicrobial resistance (AMR) is a major concern to public health and was listed among the top ten threats to global health by the World Health Organization in 2019 [1]. Due to the reduced effectiveness of antibiotics and the resulting limited treatment options, infections caused by AMR pathogens lead to prolonged illness, higher healthcare costs and increased mortality [2]. A systematic analysis estimated that 1.27 million deaths globally were attributable to bacterial AMR in 2019, including 133,000 deaths in the WHO European region [3, 4].

Among the resistant pathogens, methicillin-resistant *S. aureus* (MRSA) and vancomycin-resistant *Enterococcus faecium* (VREfm) are major contributors. The WHO therefore identifies these pathogens as a priority for research and development of new antibiotics for antibiotic-resistant bacteria [5].

*Staphylococcus aureus* is a Gram-positive, nonmotile, coagulase-positive coccoid bacterium. It is a common component of the human commensal microbiota, present in the nasal mucosa of up to 40% of the general population [6]. When skin, mucosal barriers, or the immune system are compromised, *S. aureus* can cause infection. The first description of meticillin-resistance in *S. aureus* was published in 1961 [7]. Initially, MRSA was primarily associated with healthcare settings but relevant routes of transmission in the community setting and via lifestock exposure have been documented [8]. Molecular epidemiological studies have shown that particular MRSA subtypes can be differentiated which dominate in different settings: hospital-associated MRSA (HA-MRSA), community-associated MRSA (CA-MRSA), and livestock-associated MRSA (LA-MRSA). In Germany, MRSA is one of the most important and long-standing resistant pathogens causing different hospital- and community-associated infections. Recent studies in Germany have observed a decline in its occurrence in clinical settings [9, 10].

*Enterococcus faecium* is a Gram-positive, facultative anaerobic, catalase-negative bacterium commonly found in the intestinal tract of healthy humans and animals. It is frequently associated with nosocomial infections [11]. Clinical concerns arise from its intrinsically low susceptibility to a wide range of antimicrobial agents and the emergence of vancomycin resistance, first described in 1988 [12, 13]. VREfm is one of the major emerging healthcare-associated resistant pathogens in recent years, increasing in its occurrence and resistance in Germany [14, 15].

Despite their clinical impact and status as significant contributors to bloodstream infections (BSI), the existing literature lacks a comprehensive assessment of the burden of these two pathogens, particularly in terms of Disability-Adjusted Life Years (DALYs) and attributable deaths. In our previous study, we focused on the burden of VREfm from 2015 to 2020 [16]. Building on this, we have further developed our approach to also include MRSA.

In this study we analyse and compare the magnitude of the disease burden, measured in DALYs and attributable deaths, of BSI due to VREfm and MRSA in Germany from 2017 to 2023.

We address the following research questions:

(A) How did the incidence, DALYs and attributable deaths from BSI due to MRSA and VREfm change over time?

(B) Which sex, age groups, and regions are associated with a high burden of BSI due to MRSA and VREfm?

We aim to gain a thorough understanding of the disease burden of BSI due to MRSA and VREfm, identifying potential risk groups and regions. This will provide evidence to inform public health policy, resource allocation, and targeted interventions.

Methods

### Study data

The data for this study originates from a voluntary, routine, laboratory-based surveillance system called Antimicrobial Resistance Surveillance (ARS), coordinated by the Robert Koch Institute. The study data collected by ARS covered between 508 and 662 general hospitals with information on VREfm or MRSA during the observation period, representing approximately 30% of all hospitals in Germany (Supplementary Figure S1). The data are provided retrospectively by the responsible laboratory and data status is as of 01/09/2024, after annual data validation for the previous year. ARS contributes comprehensive German surveillance data on antimicrobial resistance for a broad range of clinically relevant pathogens, including *E. faecium* and *S. aureus*, to the European Antimicrobial Resistance Surveillance Network (EARS-Net) and the Global Antimicrobial Resistance Surveillance System (GLASS). The most recent data from the surveillance system are shared in aggregated form via interactive reports on the project website [17]. Supplementary Figures S1 and S2 detail the changes in the total number of hospitals included in ARS, with the regional coverage of participating hospitals, and the distribution of hospital types in the surveillance system from 2017 to 2023 (Supplementary Figures S1 and S2).

### Variables of interest and outcome definition

A VREfm- or MRSA-positive blood culture documented in our study data is defined as a confirmed bloodstream infection of the patient. All positive hospital blood cultures were included. To estimate patient numbers from isolate information, individuals were determined in the surveillance system with a unique laboratory-specific identifier. Isolates per individual were deduplicated through the exclusion of multiple positive blood cultures per quarter for each patient. Vancomycin and methicillin resistance were defined according to the laboratories’ classifications based on the CLSI or EUCAST antimicrobial susceptibility testing guidelines. These were queried in the ARS database as follows:

- *VREfm-BSI* *E. faecium* with vancomycin resistance from blood culture
- *MRSA-BSI* *S. aureus* with oxacillin or cefoxitin resistance from blood culture

The dataset includes demographic information such as age and sex of the patient, administrative details including the date of specimen collection and the type of hospital, and geographical data indicating the federal state of the submitting hospital. The age variable was categorized into 5 year intervals, with specific groups for infants under 1 year old, children aged 1 to 4 years, and individuals over 85 years old. Geographical regions were defined according to the ARS dataset: *North West* (Bremen, Hamburg, Lower Saxony, and Schleswig-Holstein), *West* (North Rhine-Westphalia), *South West* (Baden-Württemberg, Hesse, Rhineland-Palatinate, and Saarland), *South East* (Bavaria, Saxony, and Thuringia), and *North East* (Berlin, Brandenburg, Mecklenburg-Western Pomerania, and Saxony-Anhalt).

### Burden measure

DALYs (Disability-Adjusted Life Years) measure the overall disease burden and are composed of two parts: DALY = YLL + YLD. Years of Life Lost (YLL) are years lost due to premature death from the disease or its complications, calculated using standard life expectancy figures. Years Lived with Disability (YLD) account for years lived with the disease or its sequelae, adjusted by a specific disease weighting that reflects the loss in quality of life due to the disease or its consequences. These disease weightings aim to capture the proportion of the disease’s impact on the patient’s quality of life attributable to the underlying condition or its sequelae [18].

### Burden calculation

The burden of disease for a given resistant pathogen is calculated in two steps:

1. We estimated the total annual incidence of associated bloodstream infections per 100,000 population for the time period considered based on our estimated coverage using a stratified survey sampling approach with hospitals as primary sampling units. Estimates were stratified by sex, age group, and region. The coverage of the ARS surveillance system was assessed by calculating the ratio of general hospitals that reported complete data for the entire year, to the total number of general hospitals recorded in the official hospital statistics of Germany. To address missing values in the sex variable, we applied a correction factor to adjust the incidence estimates accordingly. The incidence estimates were calculated using R (version 4.1.2) and the survey package (version 4.4) [19].
2. The estimated incidence of associated BSI were used as input for the Burden of Communicable Disease in Europe (BCoDE) toolkit. BCoDE uses an incidence- and pathogen-based approach to estimate the burden of disease for individual pathogens by simulating disease outcome trees specific to the pathogen (Supplementary Figure S4), considering both direct disease outcomes and sequelae of the infection. We used the BCoDE toolkit version 2.0.0 provided by the ECDC, performed n = 1000 simulations with no time discount, and used the life expectancy for Germany as stored in the toolkit [20]. The toolkit runs Monte Carlo simulations based on pathogen-specific disease models and incidence data, incorporating uncertainties in model parameters and incidences to produce uncertainty intervals (UIs) for our estimates (Supplementary Figure S4). The uncertainty in incidence estimates, stratified by age group and sex, was addressed by using the upper and lower boundaries of their 95% confidence intervals as the maximum and minimum of a PERT distribution. The PERT distribution is a rescaled beta distribution parametrized by the minimum and maximum value and the most probable value (in our use-case the estimated incidence) [21]. This approach allows us to estimate DALYs per 100,000 inhabitants and attributable deaths with 95% UIs. To ensure comparability with previous studies, we used the standardized life expectancy table from the Global Burden of Disease project [22, 23]. Age- and sex-stratified population numbers for Germany from 2017 were used in our analyses.

### Sensitivity analyses

For estimating the incidence, we counted all isolates submitted by each participating hospital during the 2017–2023 observation period. In the sensitivity analysis, we included only hospitals with continuous participation throughout the period to control for potential trend changes due to the included sample. This was done for the total incidence of MRSA and VREfm and stratified by region.

## Results

A total of 6262 MRSA and 5442 VREfm blood culture-positive isolates were observed within the ARS surveillance system between 2017 and 2023. The MRSA isolates were derived from 1670 females, 3417 males, and 1175 cases for which the sex was unknown. The VREfm isolates were derived from 1661 females, 3121 males, and 660 cases for which the sex was unknown. In Supplementary Figure S3 the detailed distribution of isolates across age and gender are shown for MRSA and VREfm.

### MRSA-BSI: incidence, DALYs and attributable death

**Fig. 1:**
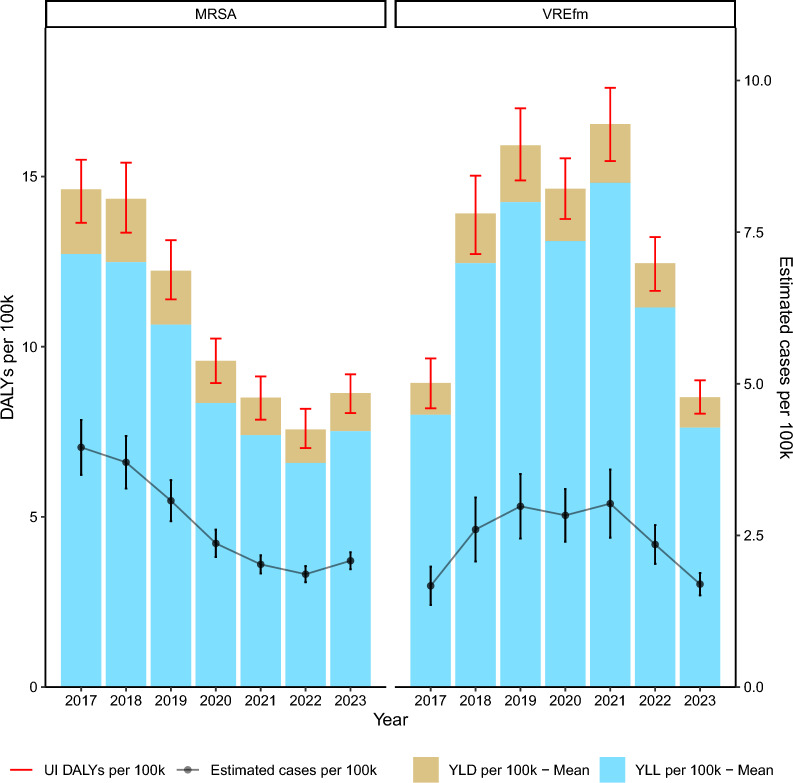
DALYs (disability-adjusted life years) per 100,000 inhabitants and estimated incidence of bloodstream infections due to methicillin-resistant *Staphylococcus aureus* (MRSA) and vancomycin-resistant *Enterococcus faecium* (VREfm) between 2017 and 2023 in Germany. YLD (years lived with disability) and YLL (years of life lost) are estimated as means of 1000 separate simulation runs. *UI* uncertainty intervals

The estimated incidence of MRSA-BSI per 100,000 inhabitants decreased from 4.0 (95% UI: 3.5–4.4) in 2017 to 2.1 (95% UI: 1.9–2.2) in 2023. After a decrease of more than 50% over this period, a slight increase of 0.2 per 100,000 inhabitants was observed in 2023 (Fig. 1).

Equally, the MRSA-BSI burden decreased from an estimated 14.6 DALYs (95% UI: 13.6–15.5) per 100,000 inhabitants in 2017 to 8.6 DALYs (95% UI: 8.0–8.6) per 100,000 inhabitants in 2023. In 2017, the number of deaths attributable to MRSA-BSI was estimated to be about 591 (0.71 per 100,00 inhabitants), which decreased to 316 (0.37 per 100,00 inhabitants) in 2023. The sensitivity analyses confirmed the overall decreasing trend of MRSA-BSI, with a slight increase in the estimated number of cases in 2023 (Supplementary Figure S4).

### VREfm-BSI: incidence, DALYs and attributable death

In contrast to MRSA, the estimated incidence of VREfm-BSI per 100,000 inhabitants increased from 1.7 (95% UI: 1.4–2.0) in 2017 to 3.0 (95% UI: 2.5–3.6) in 2021, and then slightly decreased back to 1.7 (95% UI: 1.5–1.9) in 2023 (Fig. 1).

The burden increased from 8.9 DALYs (95% UI: 8.2–9.7) per 100,000 inhabitants in 2017 to a peak of 16.5 DALYs (95% UI: 15.5–17.6) per 100,000 inhabitants in 2021, then decreased to 8.5 DALYs (95% UI: 8.0–9.0) per 100,000 inhabitants in 2023. In 2017, the number of deaths attributable to VREfm-BSI was estimated to be about 317 (0.38 per 100,00 inhabitants), which increased to approximately 577 (0.69 per 100,00 inhabitants) in 2021, and then decreased to about 327 (0.39 per 100,00 inhabitants) in 2023. The same trend was observed for VREfm in the sensitivity analyses, again with slightly higher estimated cases overall (Supplementary Figure S5).

### DALYs stratified by age and sex for MRSA-BSI

**Fig. 2:**
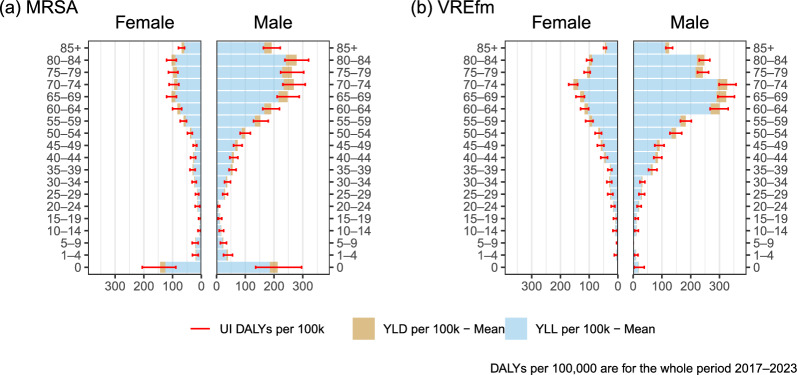
DALYs (disability-adjusted life years) per 100,000 inhabitants of bloodstream infections due to methicillin-resistant *Staphylococcus aureus* (MRSA) and vancomycin-resistant *Enterococcus faecium* (VREfm) between 2017 and 2023 in Germany. YLD (years lived with disability) and YLL (years of life lost) are estimated as means of 1000 separate simulation runs. *UI* uncertainty intervals

The distribution of DALYs per 100,000 inhabitants for MRSA-BSI from 2017 to 2023 shows variation across different age groups and between sexes (Fig. 2a). For females, the highest DALYs were observed in the age group 0–1, with a total of 143 DALYs (95% UI: 89–206) per 100,000 inhabitants in the oberserved period. This was followed by the age groups 80–84 and 65–69, with 104 DALYs (95% UI: 87–121) and 103 (95% UI: 87–121) DALYs per 100,000 inhabitants, respectively. The burden sharply decreases in younger age groups, with minimal DALYs observed in those aged 15–19. In males, the highest DALYs were observed in the age group 80–84, reaching about 278 DALYs (95% UI: 238–320) per 100,000 inhabitants. The age groups 70–74 and 75–79 follow, with 268 (95% UI: 231–308) and 262 (95% UI: 223–303) DALYs per 100,000 inhabitants, respectively. Like females, the burden in younger age groups is significantly lower, with minimal DALYs in those aged 20–24. In the 0–1 year age group MRSA-BSI present a substantial burden. For both males and females, the DALYs per 100,000 inhabitants in this age group are significantly higher than in other younger age groups. Overall, males show a higher burden than females for MRSA-BSI across all age groups.

### DALYs stratified by age and sex for VREfm-BSI

Similar to MRSA, the distribution of DALYs per 100,000 inhabitants for VREfm-BSI from 2017 to 2023 shows variations across different age groups and between sexes (Fig. 2b). For females, the highest DALYs were observed in the age group 70–74, with a total of 155 DALYs (95% UI: 140–171) per 100,000 inhabitants in the oberserved period. This was followed by the age groups 65–69 and 60–64, with 131 (95% UI: 117–146) and 116 (95% UI: 102–130) DALYs per 100,000 inhabitants, respectively. The burden decreases in younger age groups, with minimal DALYs observed in those aged 0–1. In males, the highest DALYs were in the age group 70–74, with 327 DALYs (95% UI: 299–358) per 100,000 inhabitants. This was followed by the age groups 65–69 and 60–64, with 324 (95% UI: 294–352) and 300 (95% UI: 268–331) DALYs per 100,000 inhabitants, respectively.

Overall, males show a higher burden for VREfm-BSI across all age groups than females. The burden in younger age groups is lower, with minimal DALYs in those aged 5–9. Unlike MRSA, the 0–1 year age group shows only a very small proportion of the disease burden for VREfm-BSI.

### DALY and incidence trends by region

**Fig. 3:**
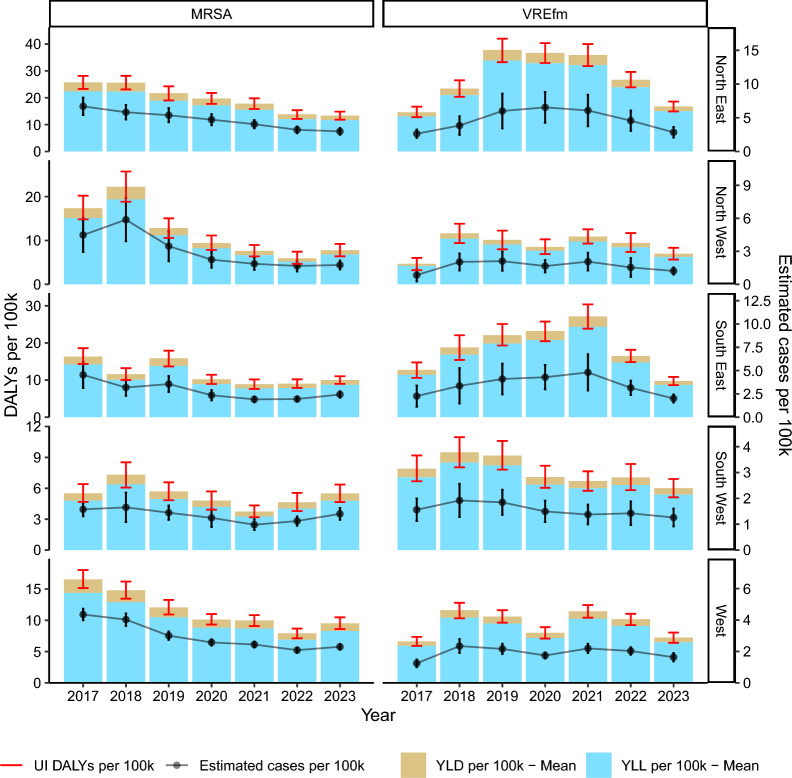
DALYs (disability-adjusted life years) per 100,000 inhabitants and estimated incidence of bloodstream infections due to methicillin-resistant *Staphylococcus aureus* (MRSA) and vancomycin-resistant *Enterococcus faecium* (VREfm) between 2017 and 2023 in Germany. YLD (years lived with disability) and YLL (years of life lost) are estimated as means of 1000 separate simulation runs. *UI* uncertainty intervals. *Note* Scales of y-axes differ between regions

There are differing trend patterns in the stratification of the DALYs and incidence measures for the defined regions (Fig. 3).

In *North East* and *South East*, MRSA-BSI show a steady decline in DALYs and incidence from 2017 to 2023. Conversely, VREfm-BSI in these regions exhibit an increasing trend in both DALYs and incidence, peaking around 2020, followed by a decline.

The *North West* and *West* demonstrate a clear and steeper declining trend in DALYs and incidence for MRSA-BSI than in *North East* and *South East*. For VREfm-BSI, these regions show an increase from 2017 to 2018, followed by a plateau in the *North West* and more variation in the *West* region. Despite this variation, both regions maintain lower levels of VREfm-related DALYs and incidence than in the *North East* and *South East.*

The region *South West* displays similar trends for the burden development of both MRSA- and VREfm-BSI, from 2017 to 2023. The DALYs and incidence for both pathogens remain relatively consistent throughout this time.

Overall, MRSA-BSI show a decreasing trend in all regions, although the extent of decrease varies. However, there is a slight increase in 2023 in all regions except in *North East*. VREfm-BSI show more regional variation, with some areas showing increases and othersmore stable trends. The different trends were confirmed by the sensitivity analyses in all regions except in *South West*, where the initial increase in VREfm-BSI in 2017 and 2018 was not observed (Supplementary Fig. 5).

## Discussion

In the study, we reported on the changing dynamics of the burden of MRSA- and VREfm-BSI from 2017 to 2023 in Germany. The incidence and DALYs due to MRSA-BSI decreased steadily from 2017 to 2021, followed by a recent levelling off in 2022 and 2023. By contrast, the incidence and DALYs due to VREfm-BSI almost doubled between 2017 and 2021, before decreasing in 2022 and 2023. The slight dip in VREfm-BSI burden in 2020 as well as the following decrease could be due to the particular circumstances in the beginning and during the COVID-19 pandemic. For example, reduction of overall patient numbers and cancelling of elective procedures may have reduced time-at-risk for vulnerable patients. Our observational study design only allows for generating hypotheses in this regard and therefore we can not differentiate whether the observed changes can be attributed causally to the influence of the pandemic or whether these changes occurred coincidentally.

While for both pathogens the burden was mainly found in the older age groups, MRSA had a larger relative burden in the highest age groups. For both pathogens, men showed higher incidences, DALYs, and attributable deaths than women, regardless of age; these differences increased with age. The higher disease burden for male individuals for both MRSA and VREfm has already been described for various antimicrobial pathogens in the literature [24]. Specifically for MRSA and VREfm, this pattern has also been observed in other countries [25, 26]. Previous studies that described higher incidences of infection with antibiotic-resistant pathogens among men came to varying conclusions: some suggested underlying biological mechanisms, others assumed gender-differences in antibiotic prescribing or poorer compliance with hand-hygiene recommendations [25, 27]. In previous analyses we could show that these gender differences can only in part be explained by higher hospitalisation rates among men [24]. To better understand the reasons for these gender differences cohort studies monitoring individual risk factors, colonisation and infection will be helpful.

The regional analysis showed clear trends, with a substantial decrease in the incidence and burden of MRSA-BSI in almost all regions, while VREfm-BSI showed more variable trends, with some regions showing particularly strong increasing trends.

In a study that focused on assessing the health burden of infections with antibiotic-resistant bacteria in the EU/EEA, there was an overall increase in the burden due to different pathogen-drug combinations from 2016 to 2020, except for MRSA [28]. In Germany, we observed a clear decrease in MRSA during this period, while the EU/EEA average experienced a stable amount of MRSA infections. The sharp increase in VREfm observed in Germany was also seen in several other European countries, such as Denmark and Ireland. The differences in prevalence and trends of VREfm observed in our study between regions were also observed between EU/EEA countries. This is consistent with the known heterogeneous distribution of VREfm [14, 29].

The age groups most affected by MRSA- and VREfm-BSI in Germany are similar to those observed in other European countries. In particular, VREfm was most prevalent in 65 to 75 year-olds, and less in the older age groups such as 80 + , while MRSA affected the age groups above 65 years to a similar extent [28]. The German population is ageing, which will lead to an increase in hospitalisations in the near future. The potential magnitude is illustrated by various projections [30]. Even when accounting for the uncertainties in the projections, the ageing population is expected to impact hospitalisation and consequently lead to an increase in the burden of infections associated with hospitalization.

In a previous study, we highlighted the occurrence in the 0–1 year age group and its significance for VREfm. In comparison to MRSA and our updated data for the calculation, this particular group was less affected and the focus shifted towards the occurrence of MRSA-BSI in the age group 0–1 year [16]. It should be noted that the estimate for the 0–1 year age group has large uncertainties due to the small sample size. However, several studies describe the relevance of BSI for the 0–1 year age group for both VREfm and MRSA [31, 32].

The sensitivity analyses conducted in this study strengthened the robustness of our findings, particularly the overall observed trends in MRSA and VREfm from 2017 to 2023. By restricting the analysis to hospitals with continuous participation from 2017 to 2023, we controlled for a potential apparent trend in the study sample introduced by variations in hospital participation over time. The consistent trends observed in both the primary and sensitivity analyses suggest that the identified incidence patterns are reliable and not substantially influenced by changes in the composition of the sample of included hospitals across the study years. However, the initial reversal trend noted in the sensitivity analysis (Supplementary Figure S6) for VREfm in the South West region in 2017 and 2018 indicated a less robust estimate for this region.

The decrease in MRSA across regions in Germany could be explained by various IPC measures and recommendations in place since 1999 by the Commission for Hospital Hygiene and Infection Prevention [33]. These recommendations were updated in 2014, highlighting the ongoing challenge of MRSA and its spread in care facilities with medical and public awareness through various studies, interventions and information [34]. The rise of VRE occurred later than MRSA, and Germany introduced less stringent recommendations for VRE prevention and control in 2018 [35].

There are notable differences in recommendations to prevent healthcare-associated infections caused by MRSA and VRE in Germany. The MRSA guidelines prioritise proactive measures, including intensive screening, adherence to strict hygiene protocols, and implementation of isolation practices across all patient groups, particularly during outbreaks. The recommendations for VRE focus more on risk assessment, considering the regional prevalence of VRE and the characteristics of the patient population. In areas with low VRE prevalence, the focus is on preventing transmission through targeted screening and isolation of colonised patients, while in regions where VRE is endemic, recommendations focus on prevention of infection in high-risk groups. Strengthening of implementation of existing MRSA prevention and control recommendations may help to further reduce MRSA burden. On the other hand for VRE, the specification of recommendations for targeted prevention and control may be further improved to support continued reduction of VRE burden in Germany. To avoid further spread, even small VRE-outbreaks should be contained effectively. It has been challenging to control prolonged VRE-outbreaks, when intervention starts with a delay. Large outbreaks with case numbers > 2000 VRE cases in Germany have been documented, contributing to regional spread [36].

Given that VRE has more recently become a public health threat in Germany, medical and public awareness appears to be lower than for MRSA, although the current burden of disease appears similarly high.

### Strengths and limitations

The study provides a robust estimate of the development of the BSI disease burden due to MRSA of VREfm in Germany from 2027 to 2023. Nevertheless, there are some limitations. The ARS surveillance system is voluntary and participation has varied over the years. Given the nature of voluntary surveillance, the data might not be entirely representative. However, our estimate calculation assumes that the data collected through the surveillance are accurate enough to draw conclusions for Germany as a whole. In particular, comparisons between regions depend on the hospital coverage of the region and the hospital levels (Supplementary Figure S1 and S2) with different case mixes included. We therefore focus on relative trends in the defined regions and absolute numbers should be interpreted with caution.

The limited number of isolates observed in some age groups, particularly in the context of outbreaks, can have a significant impact on the estimation of the burden of disease for an age group in a given year. We therefore aggregated the data by age group for the entire 2017–2023 period.

The dataset does not include clinical data. Therefore, we were not able to perform stratified analyses that included comorbidities and other patient-related information, but these factors play an important role in the occurrence of invasive infections [37]. As described in a previous publication, we used the disease outcome tree developed for vancomycin-resistant *E. faecium* and *E. faecalis* to estimate the burden of VREfm [16]. As the number of isolated *E. faecalis* from blood-cultures with confirmed vancomycin resistance was extremely low in the study data (n = 31 for the whole period 2017–2023), we decided to exlude these isolates and exclusively focus on *E. faecium* in this study.

Our study period covers the SARS-CoV-2 pandemic, and specific trends may have been influenced by differences in detection and treatment of patients. However, a meta-analysis of the incidence of different resistant pathogens, including MRSA and VRE, was unable to point in any particular direction and there is no evident indication of substantial bias [38].

Despite its limitations, our study provides, to our knowledge, the most accurate estimate and comparison of the burden of BSI due to MRSA and VREfm available, with stratification by age, sex and region in Germany. This research builds on our previous work on the burden of VREfm and adds another relevant AMR pathogen for comparison, with further expansion planned to capture the full picture of BSI due to AMR pathogens in Germany [16].

## Conclusion

Overall, we observed a clear decrease in the incidence of MRSA-BSI throughout the study period, while VREfm-BSI temporarily increased. Differences in trends and burden development from 2017 to 2023, especially for VREfm, may indicate limited awareness and undetected transmission, resulting in an often preventable serious health event. MRSA and VREfm-BSI represent a comparable burden in Germany in 2023, with high burden groups including hospitalised men over 60 years for both pathogens and neonates and infants for MRSA. Our findings highlight different high burden groups and the need for targeted prevention and continued surveillance to reduce the impact of bacterial infections and to raise awareness of potential future increases.

## Supplementary Information

Supplementary Material 1.

## Data Availability

The data analyzed in this study is available in aggregated form at https://amr.rki.de/.

## Abbreviations

AMR: Antimicrobial resistance
ARS: Antimicrobial resistance surveillance
BCoDE: Burden of communicable disease in Europe
BSI: Bloodstream infection
CLSI: Clinical and laboratory standards institute
DALYs: Disability-adjusted life years
EARS-Net: European antimicrobial resistance surveillance network
ECDC: European centre for disease prevention and control
EU/EEA: European union/European economic area
EUCAST: European committee on antimicrobial susceptibility testing
GLASS: Global antimicrobial resistance surveillance system
MRSA: Methicillin-resistant *staphylococcus aureus*
UI: Uncertainty interval
VRE: Vancomycin-resistant enterococci
VREfm: Vancomycin-resistant *Enterococcus faecium*
WHO: World health organization
YLD: Years lived with disability
YLL: Years of life lost

## Declarations

Ethics approval and consent to participate

The RKI has legal permission to process information for national surveillance of antimicrobial-resistant bacteria under §13 of the German Protection against Infection Act. Only anonymised patient data collected by hospitals and laboratories was analysed and is exempt from ethical approval and informed consent.
