# Supplementary material for: Changing dynamics of bloodstream infections due to methicillin-resistant Staphylococcus aureus and vancomycin-resistant Enterococcus faecium in Germany, 2017–2023: a continued burden of disease approach
Source: Antimicrob Resist Infect Control. 2025 Jan 30;14:4. doi: 10.1186/s13756-025-01522-9 (PMC11783909; doi:10.1186/s13756-025-01522-9)
Supplement: Supplementary file 1 — Supplementary Material 1. [file 13756_2025_1522_MOESM1_ESM.docx]

**Supplement: Changing dynamics of bloodstream infections due to methicillin-resistant Staphylococcus aureus and vancomycin-resistant Enterococcus faecium in Germany, 2017–2023: A continued burden of disease approach**

Simon Brinkwirth ^1,2,3^, Marcel Feig^4^, Ines Noll^1^, Tim Eckmanns^1^, Achim Dörre^1,2^, Sebastian Haller^1†^, Niklas Willrich^1†*^

^1^ Unit 37: Healthcare-Associated Infections, Surveillance of Antibiotic Resistance and Consumption, Department of Infectious Disease Epidemiology, Robert Koch Institute, Seestr. 10, 13353 Berlin, Germany.

^2^ Department of Infectious Disease Epidemiology, Postgraduate Training for Applied Epidemiology (PAE), Robert Koch-Institute, Berlin, Germany

^3^ ECDC Fellowship Programme, Field Epidemiology path (EPIET), European Centre for Disease Prevention and Control (ECDC), Stockholm, Sweden

^4^ Unit IT4: Development, Department of Methods Development, Research Infrastructure and Information Technology, Robert Koch Institute, Seestr. 10, 13353 Berlin, Germany.

† Shared senior authorship

*Corresponding author

Dr. Niklas Willrich

Robert Koch Institute

Department of Infectious Disease Epidemiology

Seestraße 10

13353 Berlin

Germany

WillrichN@rki.de

Table of Contents

*Distribution of hospitals in ARS study data*

Figure S1: Number of general hospitals in the Antibiotic Resistance Surveillance (ARS) included in the study data and coverage by year and region, 2017–2023, Germany

Figure S2: Distribution of healthcare level of hospitals in the Antibiotic Resistance Surveillance (ARS) included in the study data per year and region, 2017–2023, Germany

*Distribution of blood-culture isolates across age and gender*

Figure S3: Distribution of observed blood-culture isolates across age and gender, 2017–2023, Germany

*Disease outcome trees for BCoDe toolkit*

Figure S4. Disease Outcome Tree: Methicillin-resistant Staphylococcus aureus (MRSA) BSI Model /Vancomycin-resistant *Enterococcus faecalis* and *Enterococcus faecium* (VRE) BSI Model - Germany

*Sensitivtiy analyses*

Figure S5: Sensitivity analyses MRSA and VREfm of data from all laboratories compared to data from laboratories with continued participation, 2017-2023, Germany

Figure S6: Sensitivity analyses MRSA and VREfm of data from all included hospitals compared to data from hospitals with continued participation stratified by region, 2017–2023, Germany

## Distribution of hospitals in ARS study data


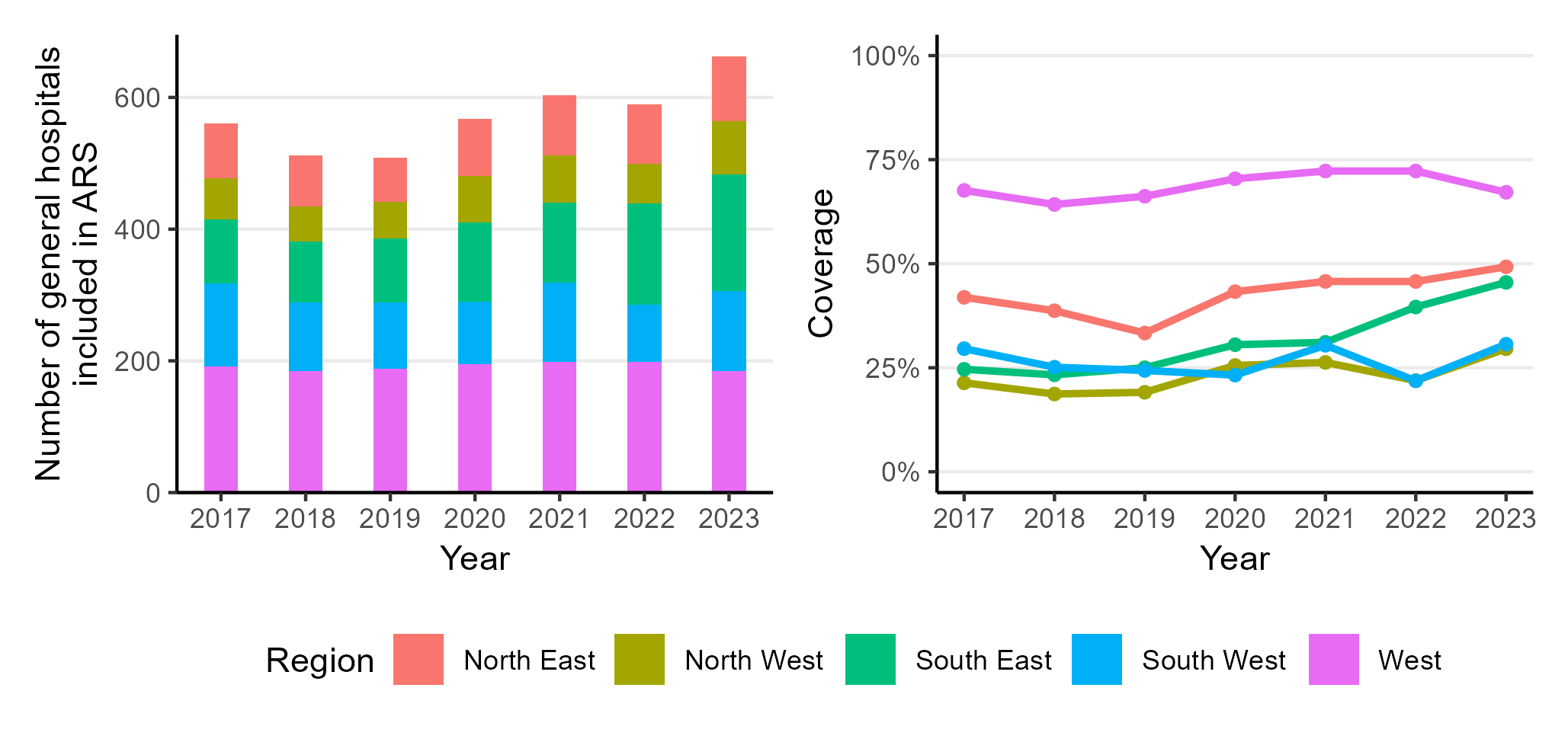


Figure S1: Number of general hospitals in the Antibiotic Resistance Surveillance (ARS) included in the study data and coverage by region, 2017–2023, Germany


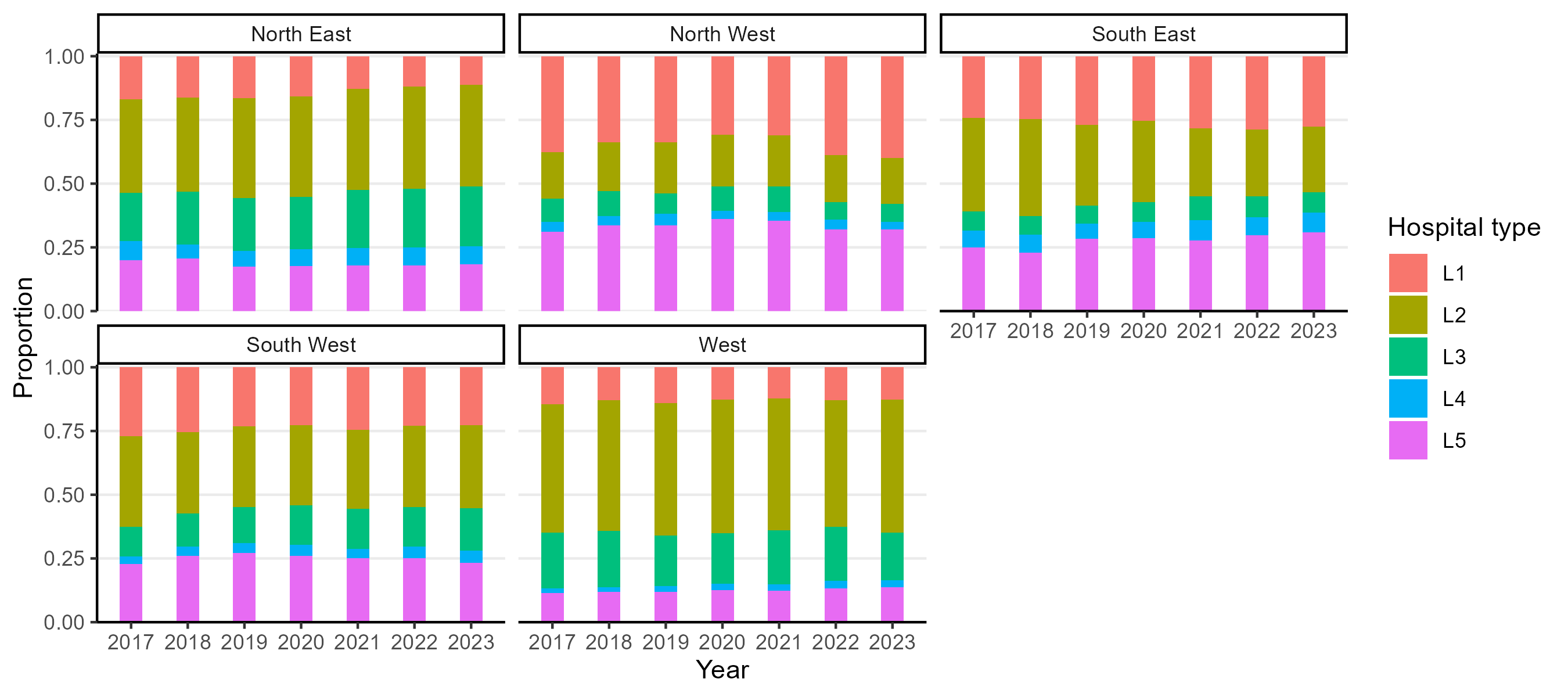


Figure S2: Distribution of healthcare level of general hospitals in the Antibiotic Resistance Surveillance (ARS) included in the study data per year and region, 2017–2023, Germany

L1—smaller general hospitals (<200 beds), L2—larger general hospitals (200–800 beds) with less than 10 specialized units, L3—larger general hospitals (200–800 beds) with more than 10 specialized units, L4—largest general hospitals (>800 beds) and university hospitals, L5—specialized hospitals (e. g. eye clinics, etc.).

## Distribution of blood-culture isolates across age and gender


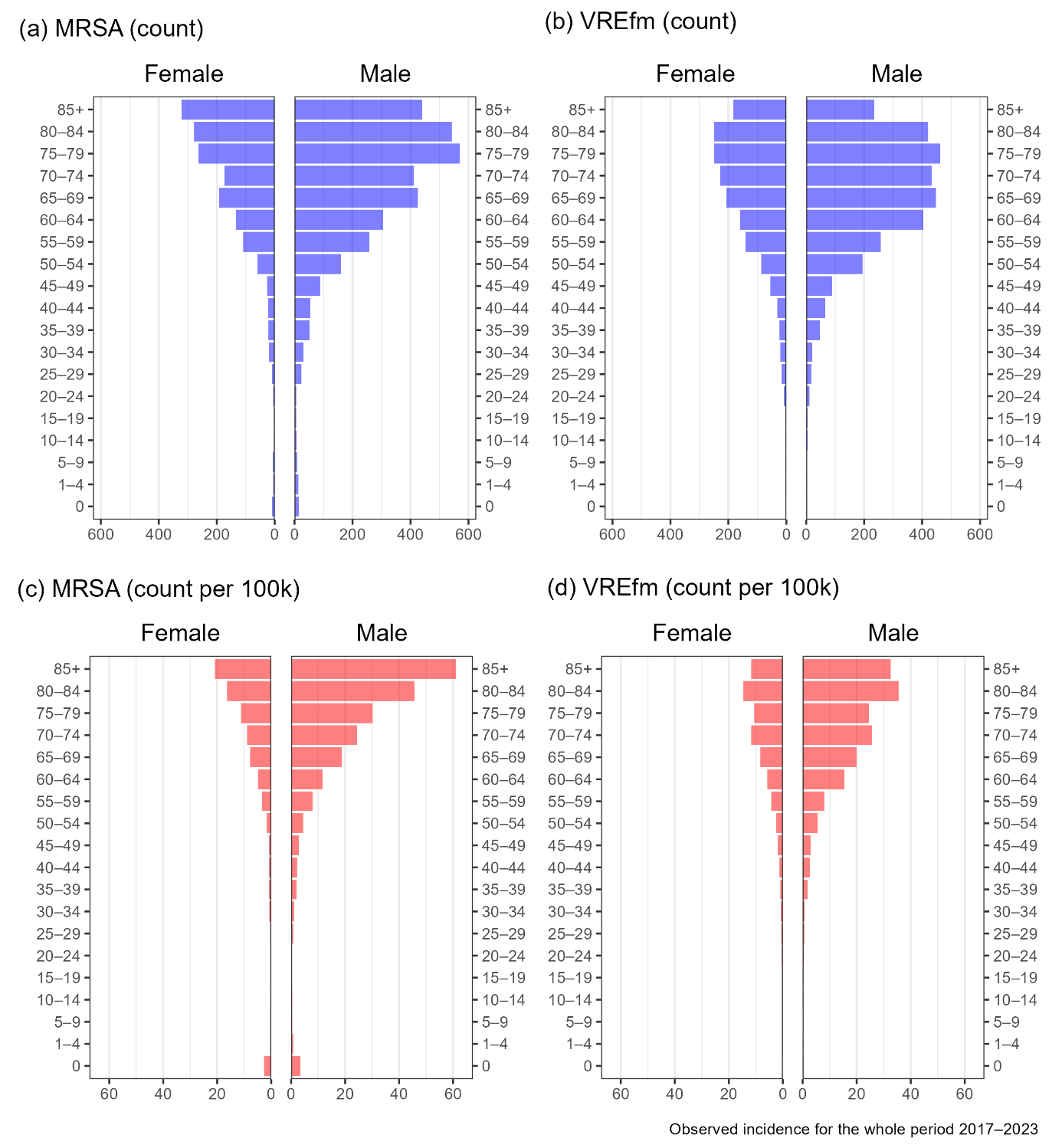


Figure S3: Distribution of observed blood-culture isolates for methicillin-resistant *Staphylococcus aureus* (MRSA) and vancomycin-resistant *Enterococcus faecium* (VREfm) in study data across age and gender, 2017-2023, Germany. In a) and b) total counts are shown and in c) and d) the counts per 100,000 inhabitants are shown.

## Disease outcome trees for BCoDe toolkit


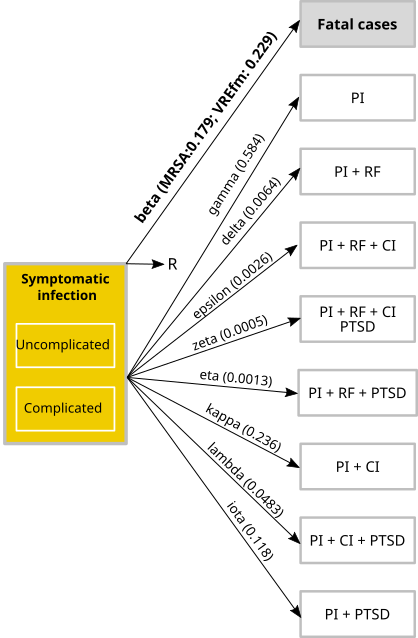


Figure S4: Disease Outcome Tree: Methicillin-resistant *Staphylococcus aureus* (MRSA) BSI Model /Vancomycin-resistant *Enterococcus faecalis* and *Enterococcus faecium* (VRE) BSI Model – Germany

CI: Cognitive impairment; PI: Physical impairment; PTSD: Post-traumatic stress disorder; R: Recovered; RF: Renal failure, renal replacement.

The most probable value for the transition probability, as determined by the PERT distribution, is shown in the outcome tree. Differences in the models for MRSA and VRE are determined by the probability of a fatal case (beta). For the complete parametrization of the transition probabilities of the outcome tree for MRSA and VRE, see (1). As there were extremely few Vancomycin-resistant *Enterococcus faecalis* bloodculture isolates and no individual model was available Vancomycin-resistant *Enterococcus faecium* (VREfm) in the BCoDE toolkit the general VRE model was used for VREfm (cf. Discussion in main text).

1. European Centre for Disease Prevention and Control. ECDC BCoDE toolkit [software application]: Version 2.0.0 2020 [Available from: https://www.ecdc.europa.eu/en/publications-data/toolkit-application-calculate-dalys].

## Sensitivity analyses


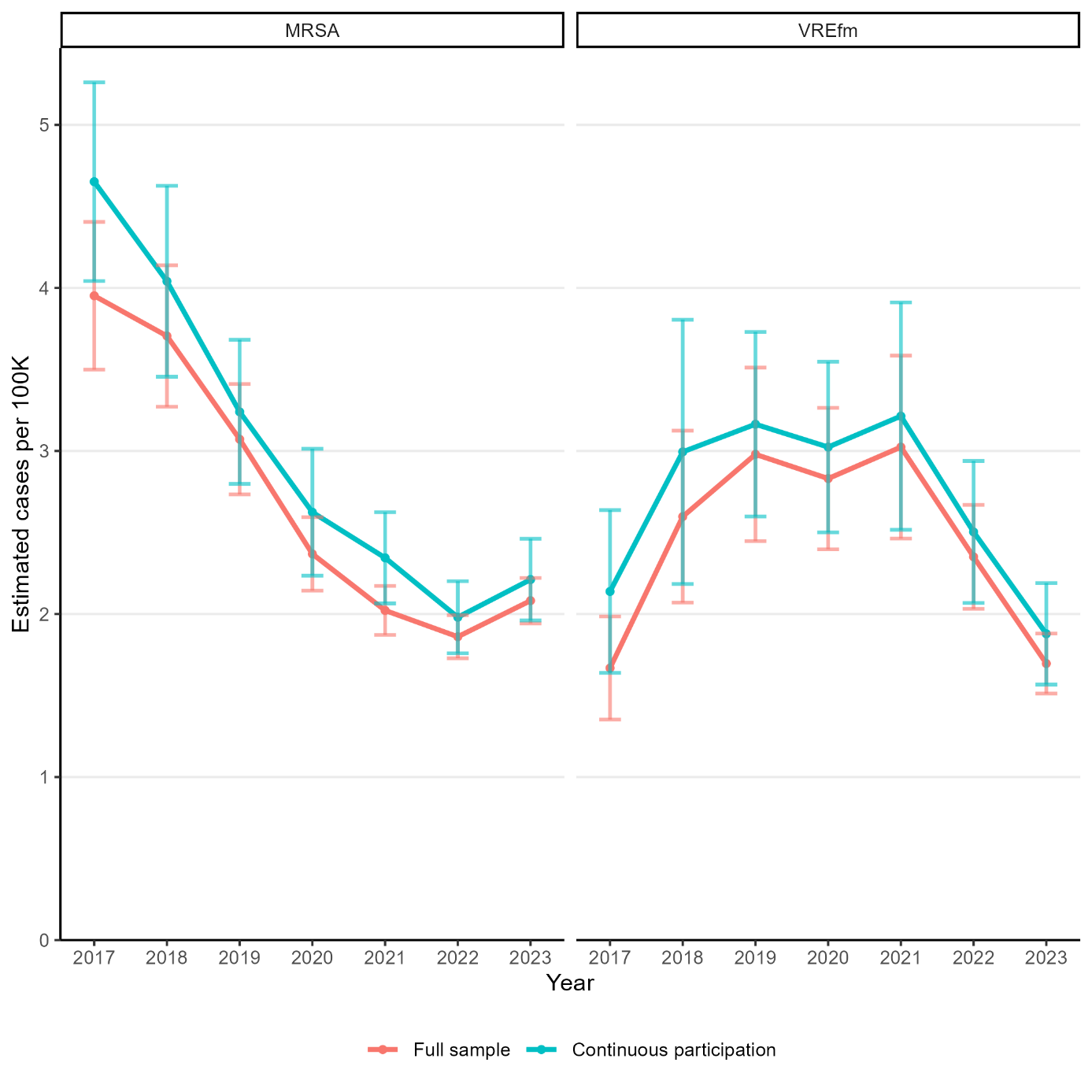


Figure S5: Results of sensitivity analysis showing estimated cases per 100,000 inhabitants for blood-stream infections with methicillin-resistant *Staphylococcus aureus* (MRSA) and vancomycin-resistant *Enterococcus faecium* (VREfm). Estimates which are based on hospitals which participated in all years of the study period (2017–2023) – labelled *Continuous Participation* – are compared with the estimates including all hospitals which participated for at least one of the years – labelled *Full Sample*.


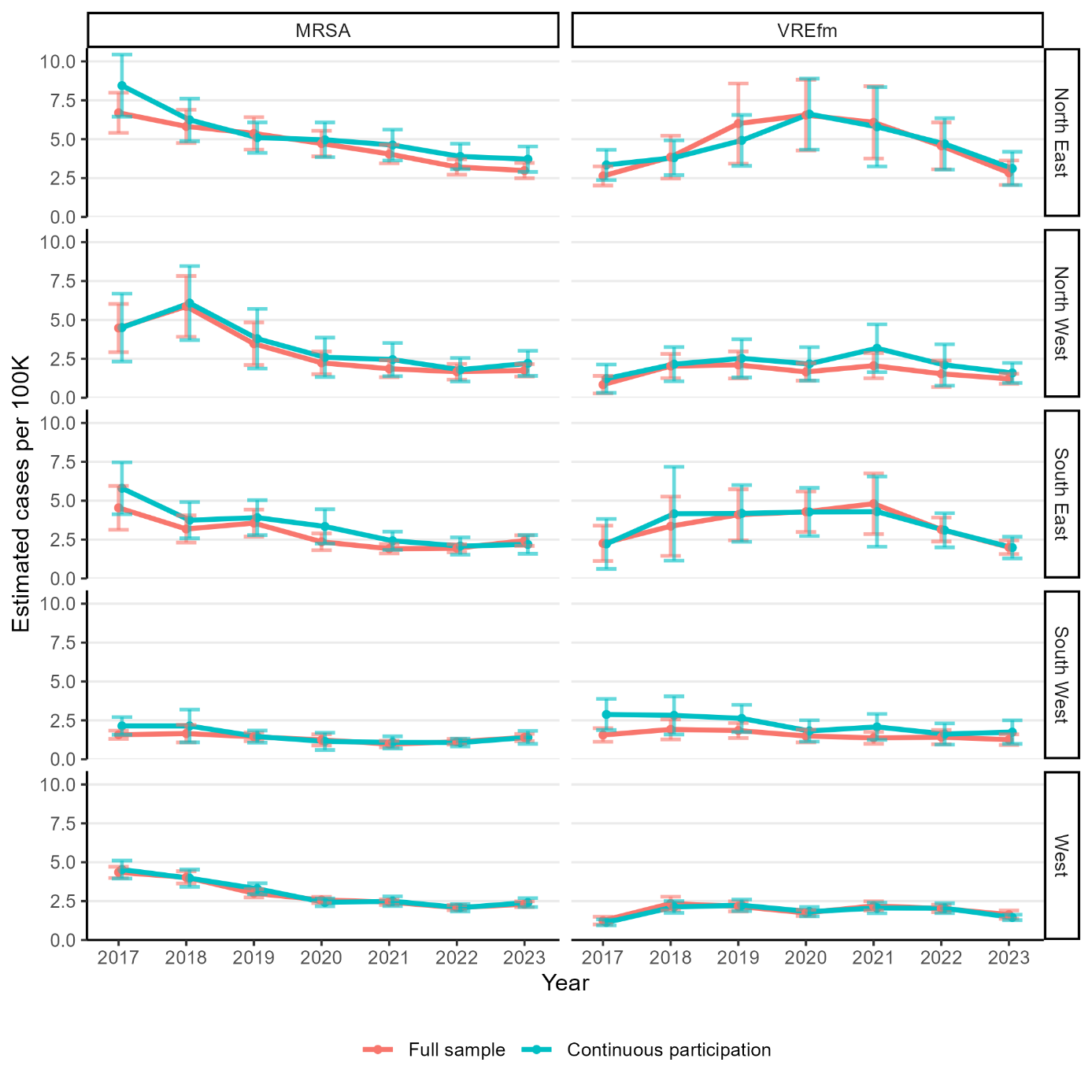


Figure S6: Results of sensitivity analysis showing estimated cases per 100,000 inhabitants for blood-stream infections with methicillin-resistant *Staphylococcus aureus* (MRSA) and vancomycin-resistant *Enterococcus faecium* (VREfm) stratified by region. Estimates which are based on hospitals which participated for all the years of the study period (2017–2023) – labelled *Continuous Participation* – are compared with the estimates including all hospitals which participated for at least one of the years – labelled *Full Sample*.
